# Genotypic and Phenotypic Characterizations of Methicillin-Resistant *Staphylococcus aureus* (MRSA) on Frequently Touched Sites from Public Hospitals in South Africa

**DOI:** 10.1155/2021/6011045

**Published:** 2021-10-23

**Authors:** Siyethaba Mkhize, Daniel G. Amoako, Christiana O. Shobo, Oliver T. Zishiri, Linda A. Bester

**Affiliations:** ^1^Biomedical Resource Unit, School of Laboratory Medicine and Medical Sciences, College of Health Sciences, University of KwaZulu-Natal, Durban 4000, South Africa; ^2^Discipline of Genetics, School of Life Sciences, College of Agriculture Engineering and Science, University of KwaZulu-Natal, Durban 4000, South Africa; ^3^Antimicrobial Research Unit, College of Health Sciences, University of KwaZulu-Natal, Durban 4000, South Africa

## Abstract

The hospital environment acts as a reservoir in the transmission of pathogens, such as MRSA, which may cause hospital-acquired infections. This study aimed to ascertain the prevalence, genetic relatedness, antibiotic resistance, and virulence profile of MRSA on some frequently touched hospital sites in South Africa. A total of 777 swabs were randomly collected from 11 frequently touched sites in the hospital environment of three wards of four public hospitals in the KwaZulu-Natal province of South Africa. Isolation of *S. aureus* and confirmation were done using genotypic and phenotypic methods. Antibiotic susceptibility testing was performed using the Kirby–Bauer disk-diffusion method. MRSA isolates were determined by the presence of the *mecA* gene. Virulence and resistance genes were detected using a standard monoplex PCR assay. ERIC-PCR was conducted to evaluate the genetic relatedness. An overall prevalence of 12.7% for *S. aureus* isolates was obtained. Out of these, 89.9% (89/99) were confirmed to be MRSA. The sites with the highest prevalence were the occupied beds (16.2% (16/99)), unoccupied beds (16.2% (16/99)), patient files (14.1% (14/99)), ward phones (13.1% (13/99)), and nurses' tables (14.1% (14/99)). The virulence genes with the highest observed frequency were *hld* (87 (87.9%)) and *LukS/F-PV* (53 (53.5%)). The resistance genes with the highest frequency were the *tetM* and *tetK* genes detected in 60 (60.6%) and 57 (57.6%) isolates, respectively. The ERIC-PCR results obtained indicated a high level of genetic diversity; however, intraclonal (within a hospital) and interclonal (between hospitals) clusters of MRSA were observed. The study showed that MRSA can contaminate various surfaces, and this persistence allows for the dissemination of bacteria within the hospital environment. This highlights the need for improved infection prevention and control (IPC) strategies in public hospitals in the country to curb their potential transmission risks.

## 1. Introduction

A hospital-acquired infection (HAI) or nosocomial infection develops during hospitalisation or within 48 hours after the patient has been discharged. In most cases, this is not the initial cause of hospital admission [1]. *Staphylococcus aureus* (*S. aureus*) is considered one of the most important [2] pathogens responsible for HAIs. HAIs are a financial burden in developed and developing countries causing significant strain on the economy due to the high cost of treatments and increased mortality and morbidity rates that are associated with these types of infections [3–5]. In addition, *S. aureus* is one of eight significant pathogens listed by the Global Antimicrobial Resistance Surveillance System (GLASS) alongside *Shigella* spp., *Salmonella* spp., *Streptococcus pneumoniae*, *Klebsiella pneumoniae*, *Neisseria gonorrhoeae, Acinetobacter* spp., and *Escherichia coli* [6].

*S. aureus* is a versatile pathogen that causes a variety of diseases, from respiratory to skin infections [7], as it colonises the skin and mucosal membranes of animals and humans [8]. Lung infections are predominantly of nosocomial origin. In contrast, skin infections are primarily of a community-acquired nature [7]. The severity of the disease ranges from minor skin infections to life-threatening conditions (soft tissue abscesses, toxic shock syndrome, pneumonia, septicaemia, bacteraemia, and endocarditis) [9]. *S. aureus* strains have become resistant to a diverse range of antibiotics resulting in multidrug-resistant (MDR) strains. There is an increasing shortage of antibiotics that can be used to treat these infections [10]. Methicillin is one of the antibiotics used to treat *S. aureus* infections; the term MRSA is used for methicillin-resistant *S. aureus* strains. MRSA is a global multidrug-resistant human pathogen present in hospital and community environments [11].

Environmental sampling of the hospital surfaces indicated that ESKAPE (*Enterococcus faecium*, *Staphylococcus aureus*, *Klebsiella pneumoniae*, *Acinetobacter baumannii*, *Pseudomonas aeruginosa*, and *Enterobacter* spp.) pathogens are shed into the hospital environment by patients and survive on surfaces for an extended period as they are difficult to destroy through cleaning and disinfection [12]. Within the hospital, MRSA has been isolated from hospital equipment in Ireland [13], hospital linen in France [14], telephones in Nigeria [15], and air [16]. However, there is a dearth of information on the prevalence and characterization of MRSA within the hospital environments in South Africa. Therefore, there is a need to determine if current infection, prevention, and control (IPC) strategies are adequate to curtail MRSA in the hospital environment of public hospitals. Given this information, the aims of this study were two-fold: firstly, to examine the prevalence of *S. aureus* and MRSA on inanimate surfaces in South African public hospitals; secondly, to determine the genetic diversity, antibiotic resistance, and virulence profiles of the collected isolates.

## 2. Materials and Methods

### 2.1. Sample Site

Swab samples were collected over three months from September to November 2017. Four provincial public hospitals are classified under the South African National Health Act of 2003 [17] according to their different levels of healthcare viz. central (hospital A), tertiary (hospital B), regional (hospital C), and district (hospital D). Central/specialized hospitals, with bed sizes between 800 and 1200, accept referrals from both district and regional hospitals. Tertiary hospitals have 400 to 800 beds available and provide specialised services such as those offered by a regional hospital. They also receive referrals from regional hospitals; however, referrals are not limited to provincial boundaries. Regional hospitals which have between 200 and 800 beds receive referrals from several district hospitals and provide services to a specific regional population. District hospitals have between 50 and 600 beds, depending on their classification of either being small, medium, or large. These hospitals receive outreach and support from specialists based at regional hospitals. Samples were collected from three wards viz. intensive care unit (ICU) and paediatric and general wards of the studied hospitals.

### 2.2. Sample Collection

A total of 777 swabs were collected from eleven predetermined and frequently touched surfaces in the hospital environment viz. ward phones, nurses' tables, drip stands, sinks, blood pressure (bp) machines, patient files, occupied and unoccupied beds, ventilators, mops, and the utility room door handles (Supplementary Table S1). The stratified random sampling method was used. Samples were collected by gently swabbing approximately a 5 cm circumference of the surfaces using capped Amies Agar swabs in translucent transport media (Thermo Fisher Scientific, Waltham, MA, USA). The swabs were then placed in a cooler box with ice for transportation to the laboratory and processed within four hours upon arrival.

### 2.3. Phenotypic Identification and Isolation of *Staphylococcus aureus*

For enrichment purposes, swabs were broken off into labelled 50 mL blue-capped centrifuge tubes containing 20 mL of tryptic soy broth (TSB) (Sigma-Aldrich, Germany) and incubated at 37°C for ± 24 hrs while stirred (Steyn Scientific, USA). After incubation, a loopful of broth was aseptically inoculated and streaked onto plates containing chromogenic agar, HiChrome^TM^*Aureus* Agar Base (HiMedia, India), and supplemented with 2% egg-yolk tellurite emulsion (HiMedia, India). The plates were incubated (Shel Lab, Sheldon Manufacturing Inc., USA) at 37°C for ± 24 hrs. The presumptive *S. aureus* colonies were subsequently stored in cryovials containing TSB supplemented with 10% glycerol (VWR International Life Sciences, Amresco, Parkway) until further analyses.

### 2.4. Deoxyribonucleic Acid (DNA) Extraction

Total genomic DNA was extracted using the conventional boiling method [18]. Briefly, colonies from pure *S. aureus* cultures were suspended in 300 *μ*L of TE (Tris-EDTA) (10 mM Tris-HCl pH 8.0 with 1 mM EDTA) buffer and vortexed to homogenise the cells. The suspension was boiled in a heating block at 100°C for 10 minutes with immediate cooling on ice for 5 minutes. The tubes were centrifuged (Beckman Coulter Microfuge 16, United States) at 14000 ×*g* for 5 minutes. The supernatant was transferred into a new sterile tube and stored at −20°C for PCR use. Nanodrop readings were taken to measure the concentration and purity of isolated DNA using the Thermo Scientific Nanodrop 2000, UV-Vis spectrophotometer (Wilmington, Delaware, USA). Readings within 1.8–1.9 at the ratio of 260/280 were regarded as pure DNA. Nuclease-free water (Thermo Fisher Scientific, Waltham, MA USA) was used to adjust the DNA concentration accordingly for PCR.

### 2.5. Molecular Confirmation of *S. aureus*

Primers for the polymerase chain reaction (PCR) were synthesised at Inqaba Biotechnology (South Africa). The primer sequences, annealing temperatures, and product sizes are indicated in Table 1. The species-specific thermonuclease *nuc* gene [19] was used for the identification of *S. aureus.* The positive control *S. aureus* ATCC 25913 and negative control (nuclease-free water) were used for all reactions. The Bio-Rad T100^TM^ Thermal Cycler (Singapore) was used to carry out the PCR assays. A 15 *μ*L reaction contained 7 *μ*L of DreamTaq Green PCR Master Mix (2X) (Thermo Fisher Scientific, Waltham, MA, USA), 0.5 *μ*L of both reverse and forward primers of a 20 *μ*M primer concentration, 3 *μ*L of template DNA, and 4 *μ*L of nuclease-free water making a total reaction volume of 15 *μ*L. Gel electrophoresis was carried out by loading the PCR products into a 1.5% (w/vol) agarose gel with 5 *μ*L of 10 mg/ml ethidium bromide (Thermo Fisher Scientific, Waltham, MA, USA) and run at 100 volts for 30 minutes in a tank containing Tris-borate-EDTA (pH 8.3, 1X). A 100 bp DNA ladder (New England Biolabs, Ipswich, USA) was used as the molecular weight marker. Gels were viewed on the ChemiDoc^TM^ Imaging System (Bio-Rad Laboratories Inc., USA).

### 2.6. Antimicrobial Susceptibility Testing of Isolated Bacteria

Antimicrobial susceptibility testing was performed using the Kirby–Bauer disk-diffusion method, according to the Clinical and Laboratory Standards Institute M100-S27 guidelines [23], using Mueller–Hinton agar (Oxoid, England). An inoculum was suspended in sterile water and developed using the 0.5 McFarland standard (DEN-1B McFarland, Biosan, Latvia). All antibiotics were sourced from Oxoid, England. The following antibiotics were used: erythromycin (macrolides) (15 *μ*g), cefoxitin (penicillins) (30 *μ*g), penicillin (penicillins) (10 units), tetracycline (tetracyclines) (30 *μ*g), linezolid (lipopeptides) (30 ug), clindamycin (lincosamides) (2 *μ*g), quinupristin-dalfopristin (streptogramins) (15 *μ*g), ciprofloxacin (fluoroquinolones) (5 *μ*g), and rifampicin (ansamycins) (5 *μ*g). These antibiotics were selected based on a review conducted by Gold and Pillai (2009) for their clinical use in human therapy. *S. aureus* ATCC 25923 was used as the control strain. Isolates were considered multidrug resistant (MDR) when they showed resistance to at least one antibiotic in three or more different antibiotic classes [24].

### 2.7. Genotypic Identification of Resistance and Virulence Genes

DNA extraction and volumes used to conduct singleplex reactions for the resistance and virulence genes were as described previously (Table 1). The PCR primers and conditions followed are indicated in Table 1. The resistance genes, *aac* (6′)*-aph* (2″), *blaZ*, *tetK*, *tetM*, and *ermC*, which confer resistance to aminoglycoside, macrolide-lincosamide-streptogramin B (MLS_B_), tetracycline, and erythromycin, respectively, were identified. Virulence genes for Panton–Valentine leucocidin (*LukS/F-PV*) and alpha and delta haemolysin (*hla* and *hld*) were also identified. The positive control *S. aureus* ATCC 25913 and negative control (nuclease-free water) were used for all the reactions conducted.

### 2.8. Analysis of the Genetic Diversity of *S. aureus* Using Enterobacterial Repetitive Intergenic Consensus (ERIC) PCR

Genomic DNA was extracted using the Quick-DNA^TM^ Miniprep Plus kit (Zymo Research, USA) according to the manufacturer's instructions. The purity and concentration were measured using the Thermo Scientific Nanodrop 2000, UV-Vis spectrophotometer (Wilmington, Delaware, USA). A reaction volume of 25 *μ*L was set up: 12.5 *μ*L of DreamTaq Green PCR Master Mix 2X (Thermo Fisher Scientific, Waltham, MA, USA), 9.3 *μ*L of nuclease-free water, 3 *μ*L of template DNA, and 0.1 *μ*L of 100 mM ERIC 1 (5′-CACTTAGGGGTCCTCGAATGTA-3′) and ERIC 2 (5′-AAGTAAGTGATGGGGTGAGCG-3′) primers (Inqaba Biotechnology, South Africa). The PCR conditions were as follows: 95°C for 2 minutes, 35 cycles of 30 s of denaturation at 90°C, 1 min of annealing at 52°C, 8 min of extension at 65°C, and final elongation at 65°C for 16 min. Gel electrophoresis was performed at 70 V for 75 min. The ERIC-PCR gels were captured (ChemiDoc^TM^ Imaging System (Bio-Rad Laboratories Inc., USA) and analysed using BioNumerics software version 6.6 Applied Maths NV (bioMérieux, Sint-Martens-Latem, Belgium). A dendrogram was produced using an unweighted pair group with arithmetic mean (UPGMA) method and Dice coefficient parameters of 1% tolerance and 0.5% optimisation.

### 2.9. Statistical Analysis

SPSS software version 25 (IBM SPSS Statistics) was used for statistical analyses using a probability value of less than 0.05 (*p* < 0.05) for significance. Fisher's exact and Pearson's chi-square tests were used to determine the significance of the relationship between the genes detected and the hospital/site/wards from which the samples originated. For each statistical model, the dependent variable was the virulence gene presence (0 = absent; 1 = present).

## 3. Results

### 3.1. Prevalence of *Staphylococcus aureus*

**Figure 1:**
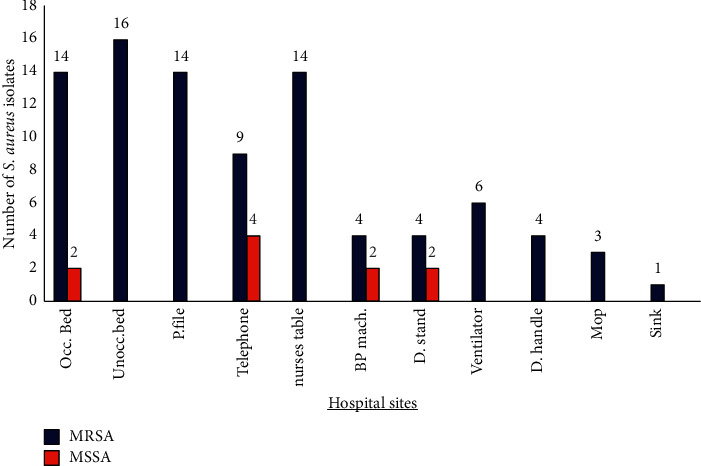
Prevalence of *S. aureus* (MRSA and MSSA) isolates sampled on eleven hospital sites within the wards and public hospitals investigated.

From the 777 samples collected, a total of 99 (12.7%) *S. aureus* isolates were obtained over a period of three months. The prevalence of *S. aureus* isolates per hospital was as follows: the central hospital (hospital A) had a majority of 23.2% (23/99), the tertiary hospital (hospital B) had a prevalence of 23.2% (23/99), the regional hospital (hospital C) had a prevalence of 33.3% (33/99), and the district hospital (hospital D) had a prevalence of 20.2% (20/99). With regard to the wards, the general ward revealed the highest prevalence with 41.4% (41/99) isolates, followed by the paediatric ward (34.3% (34/99)) and ICU (24.2% (24/99)); no statistical significance was observed (*p* ≥ 0.05). Isolates were recovered from all the eleven sites sampled. The sites with the highest prevalence (indicated in Figure 1) were the occupied beds (16.2% (16/99)), unoccupied beds (16.2% (16/99)), patient files (14.1% (14/99)), ward phones (13.1% (13/99)), and nurses' tables (14.1% (14/99)). The sites with the lowest prevalence were the blood pressure apparatus (6.1% (6/99)), drip stands (6.1% (6/99)), ventilators (6.1% (6/99)), door handles (4% (4/99)), mops (3.0% (3/99)), and sink (1.0% (1/99)). The *mecA* gene was identified in 89.9% (89/99) of the *S. aureus* isolates. Only ten isolates were identified as methicillin-susceptible *S. aureus* (MSSA) (Figure 1). There was no statistical significance (*p* > 0.05) observed between the presence of the *mecA* gene and the hospital levels (Table S2).

### 3.2. Antibiotic Susceptibility Tests

Antimicrobial susceptibility testing was performed on all the 99 isolates collected. The isolates showed the highest resistance to penicillin (60, 60%), followed by cefoxitin (46, 46%) and erythromycin (42, 42%). Less resistance was observed for quinupristin-dalfopristin (29, 29%), clindamycin (27, 27%), ciprofloxacin (18, 18%), tetracycline (10, 10%), rifampicin (13, 13%), and linezolid (3, 3%). A total of 24/99 (24%) of the isolates were multidrug resistant (MDR). Three isolates were resistant to six of the antibiotic classes: central hospital (A) (ward phone in paediatric wards), regional hospital (C) (ward phone in the ICU), and district hospital (D) (ventilator in the ICU).

### 3.3. Virulence and Resistance Genes

The virulence genes with the highest observed frequency were *hld*, haemolysin gene (87 (87.9%)), and *LukS/F-PV (*53 (53.5%)) encoding for the Panton–Valentine leucocidin gene. The *hla* (haemolysin) gene had the lowest frequency of 29 (29.3%). The resistance genes with the highest frequency were the *tetM* and *tetK* genes encoding tetracycline resistance which were detected in 60 (60.6%) and 57 (57.6%) of the isolates, respectively. The *blaZ* and *ermC* genes, encoding *β*-lactamase and erythromycin resistance, were present in 53 (53.5%) of the isolates. The *aac* (6′)-*aph* (2″) gene had the lowest frequency of 29 (29.3%).

Pearson's correlation analyses (Table S3) demonstrated that the *mecA* gene had a significant (*p* < 0.05) positive correlation with the *tetK* (25.5%), *ermC* (36.0%), *blaZ* (22.5%), *aac* (6′)-*aph* (2″) (21.6%)*, tetM* (41.6%), and *LukS/F-PV* (22.5%) genes. The study also identified that the *aac* (6′)-*aph* (2″) gene had a positive correlation (*p* < 0.05) to *tetM* (24.6%)*, tetK* (28.3%), *ermC* (33.3%), *blaZ* (37.7%), and *LukS/F-PV* (19.9%) genes. The *blaZ* gene had a significant positive correlation (*p* < 0.05) with *ermC* (47.2%) and *tetM* (20.2%). The *tetK* gene had a strong and positive correlation (*p* < 0.05) with *ermC* (30.7%), *hla* (24.3%), *tetM* (35.4%), and *LukS/F-PV* (34.8%). *ermC* had a strong and positive correlation (*p* < 0.05) with *tetM* (36.8%) and *LukS/F-PV* (39.1%) (Table S3).

The relationships between the genes and site, genes and ward, and genes and hospital were examined. Pearson's chi-square and Fischer's exact test indicated that there was a significant relationship (*p* < 0.05) between the *mecA* gene and the site. A significant relationship (*p* < 0.05) was identified between the hospital and the *tetK*, *ermC*, *aac* (6′)-*aph* (2″), and *LukS/F-PV* genes (Table S2).

### 3.4. Enterobacterial Repetitive Intergenic Consensus Sequence-PCR (ERIC-PCR) of MRSA

**Figure 2:**
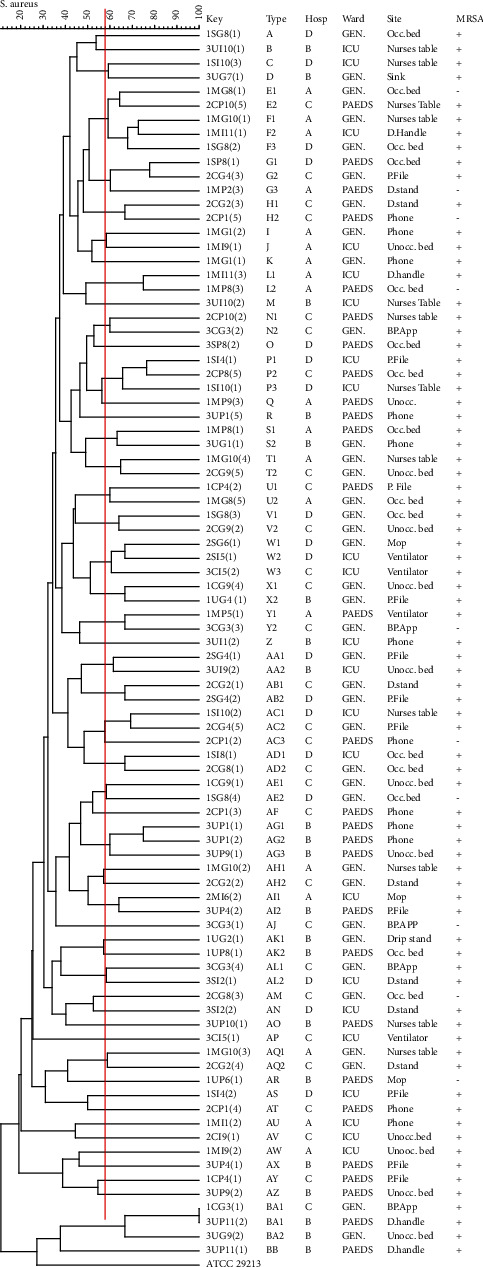
ERIC-PCR fingerprinting of MRSA ERIC-type groups of isolates recovered from four public hospitals in KZN, South Africa. An index of 60%, indicated by the solid red line, was used, respectively, to determine similarity and clustering. *S. aureus* ATCC 29213 was used as the quality control strain.

The enterobacterial repetitive intergenic consensus (ERIC-PCR) was used to determine the genetic diversity of MRSA isolates. Bands were produced for 87 of the isolates recovered; 2 of the isolates were nontypeable (these isolates showed no bands under ERIC-PCR conditions). The 87 isolates were assigned to 54 different ERIC types, namely, A-BC, based on a similarity index of ≥60% (shown by the solid red line) (Figure 2). The results obtained indicated a high level of genetic diversity in the study isolates. However, it was observed that 21% (18/87) of the *S. aureus* isolates were grouped into six major ERIC types: F (*n* = 3), G (*n* = 3), P (*n* = 3), W *n* = 3), AC (*n* = 3), and AG (*n* = 3) (Figure 2). Of these six main ERIC types, three major ERIC types (P, W, and AC) were shared between different two hospitals, viz. district hospital (D) and regional hospital (C), indicating a potential interclonal spread of MRSA. More so, the AG (*n* = 3) cluster indicated that isolates from the ward telephone (*n* = 2) and unoccupied bed (*n* = 1) within the paediatrics ward of the tertiary hospital (B) belonged to the same ERIC type, also suggesting a possible intraclonal spread of MRSA.

## 4. Discussion

The sanitation of a hospital environment plays a crucial role in spreading pathogenic organisms such as MRSA [25]. MRSA can be transferred from person to person or from person to frequently touched objects in the hospital environment, and vice versa [15]. The overall prevalence of *S. aureus* obtained was 12.7% (99/777) which comprised 89 isolates of MRSA (methicillin-resistant *S. aureus*) and ten isolates of MSSA (methicillin-susceptible *S. aureus*). The result obtained indicated a low prevalence of MSSA. Although the reported MSSA prevalence was low, MRSA and MSSA do not differ in the diseases they cause [26]. In Africa, a lower prevalence rate of 2.7% (1/37) was reported by Adekunle et al. [27], who had studied environmental isolates collected from a general hospital in Nigeria. In addition, a prevalence of 17% (8/47), mainly from door handles, was reported in a study conducted in three government hospitals in Ghana [28].

The prevalence rate obtained in this study was higher than a survey conducted by Mukhiya et al. [29], who obtained 40.7% (11/27) from environmental isolates collected from hospitals in Nepal, a developing country. The level of healthcare provided by these hospitals had not been specified. The prevalence in our study was also higher than that of Ekrami et al. [30], who obtained an MRSA prevalence of 60.0% from hospital environmental isolates collected from hospitals in Iran which is also a developing country. South African public hospitals lack funding, have a shortage of resources, are understaffed, and are often overcrowded [31]. These factors greatly affect IPC implementation and may contribute to a lack of hospital hygiene management. The regional hospital (hospital C) had the highest number of isolates compared to the other three hospitals. However, patients may act as vectors that translocate hospital-acquired pathogens between hospitals [32]. This was evident in a study conducted by Donker et al. [32], who reported a positive correlation (33.0%) between patient referrals and the incidence of hospital-acquired pathogens such as MRSA in hospitals in England and the Netherlands.

One of the transmission routes of MRSA is through direct skin contact and shedding of epidermal skin cells [33]. Infected patients or patients that are carriers of MRSA may shed their skin onto the hospital beds. The results also revealed that there was a significant relationship (*p* < 0.05) between the *mecA* gene and the site (shown in Table S2). These results are similar to those of Adwan et al. [34], who also reported a significant relationship. It is interesting to note that the highest prevalence of MRSA was obtained from the unoccupied beds compared to other sites in the study (Figure 1). These results are an indication that IPC protocols pertaining to the laundry or hospital bed disinfection were unsatisfactory as the presence of MRSA may have emanated from a previously admitted patient. The results showed that the occupied and unoccupied beds accounted for 32.4% of the total isolates. These results indicate substandard cleaning agents or improper execution of IPC protocols. Contamination of unoccupied beds may occur before the patient has had direct contact with the site. These results are consistent with the results obtained by Pinon et al. [14], who conducted a study in a hospital in France as *S. aureus* has been reported to contaminate bed linen even though the sheets and pillowcases had been washed [14]. A possible reason may be attributed to the survival abilities of *S. aureus* for one to 90 days or more on fabrics and materials such as cotton, cotton terry, cotton-polyester blend, polyester, and polypropylene plastic [35].

*S. aureus* has been associated with a low infectious dosage indicating that *S. aureus* is highly contagious even in small amounts; only 15 cells of *S. aureus* introduced into experimental lesions were enough to result in infection [36]. Therefore, patients through direct contact with the surface may be exposed to the pathogen through open wounds or postsurgical procedures, thus suggesting that contaminated surfaces may be an essential and underappreciated source of MRSA transmission [33]. Transmission of pathogens is dependent on a range of factors which are but not limited to the viability of the pathogen on that environmental site, relative humidity, the frequency of contact between patients, healthcare workers, and contaminated surfaces, ambient temperature, and the dose of the transmitted pathogen [34].

The antibiotic susceptibility testing results showed that only 46% of the isolates were resistant to cefoxitin, the antibiotic used as an indication for methicillin resistance of *S. aureus* (MRSA) isolates, according to CLSI recommendations (M100-S27, 2017). The results obtained were contrary when compared to the high *mecA* gene presence of 89% indicated by the PCR results. *S. aureus* strains that are methicillin resistant carry either the *mecA*, *mecB*, or *mecC* genes. These genes are acquired genetic determinants that encode for PBP2a or PBP2a′, low-affinity penicillin-binding proteins [26]. PBP2a has a very low affinity for most *β*-lactam antibiotics [26].

The level of *mecA* transcription or presence in isolates does not predict the level of phenotypic methicillin resistance. A possible explanation is found in the work of Lee et al. [37], who identified three attributing factors. The first factor is stringent stress response (the bacteria's reaction to different stress conditions, such as amino acid, fatty acid, iron limitation, and heat shock) [37]. The work of Boyle-vavra et al. [38] presented the second factor. The inactivation of *VraS* was shown to have induced the transcription of *mecA* but did not increase the level of PBP2a activity [38]. *VraS* is a part of the regulatory system made up of the sensor protein *VraS* and response regulator protein *VraR*. These proteins are involved in controlling the cell wall peptidoglycan biosynthesis [37]. The third factor is the chaperone foldase protein, *PrsA,* which changes the amount of correctly folded PBP2a that is found in the membrane. As a result, this would in turn affect the methicillin resistance without hindering the transcription of the *mecA* gene. Thus, these factors are an indication that the gene presence does not determine antibiotic resistance [37].

The presence of the *LukS/F-PV* gene is commonly associated with strains of community-acquired MRSA; however, this varies based on geographic locations [39]. The virulence gene for Panton–Valentine leucocidin, *LukS/F-PV,* was detected in 53.5% of the isolates. This percentage was higher than that previously reported by Adwan et al. [34], who obtained an incidence of 14.3% in a similar study. The presence of this gene indicates the possible production of a toxin, which induces the formation of virulence pores in leukocytes [34]. If patients are exposed to these strains, this may result in severe chronic skin infections or necrotising pneumonia with an extremely high mortality rate even in young and healthy patients. The presence of the *LukS/F-PV* gene obtained in this study is contrary to the results of Bhatta et al. [39], who reported that there was no presence of this gene among hospital environment isolates collected in Nepal, which may have been an indication of the gene not being associated with isolates from the hospital environment.

There are limited studies that have implemented ERIC-PCR to evaluate the clonality of *S. aureus* isolates from the hospital environment. The ERIC-PCR results obtained in this study showed a high diversity between the MRSA isolates collected. The results showed that 2 of the isolates were nontypeable with ERIC-PCR. These results were comparable to the study conducted by Adwan et al. [34] whose results indicated that two isolates out of 265 swabs collected from two hospitals in Iran were nontypeable using ERIC-PCR. The ERIC-PCR results indicated that three of the six major ERIC types were shared between the district hospital (D) and regional hospital (C) indicating a potential interclonal spread of MRSA clones as district hospitals fall under level 1 of the referral system of South African hospitals [40]. Patients are referred from the district hospitals to the local and regional hospital whenever the correct health service cannot be offered. These results further highlight the transmission risk of pathogens between hospitals due to referrals. The spread of MRSA was also observed between two sites of the same hospital ward (Figure 2) suggesting an intraclonal spread of MRSA. The failure to adhere to IPC measures is a likely cause. However, the study was limited by the lack of information on the infection control practices at these hospitals that precluded the comparative analyses of the IPC practices between different hospital settings, warranting further studies to ascertain this.

## 5. Conclusion

In summary, the study highlights the prevalence and phenotypic and genotypic characterizations of *S. aureus* indicating that the hospital environments could act as potential reservoirs in the transmission of MRSA encoding diverse antibiotic resistance and virulence genes in South African public hospitals. This was further supported by ERIC-PCR typing showed by the recovery of genetically similar MRSA isolates from different surfaces within the same hospital (intraclonal spread) and between different hospitals (interclonal spread), which can be further disseminated to other sites if IPC measures are suboptimal. We conceive that our work can be used as a framework for future surveillance initiatives to improve hospital hygiene through IPC-centred strategies to minimize the presence of drug-resistant and pathogenic microorganisms that are present in the hospital environment. This would reduce hospital-acquired infections, due to contaminated sites, and provide a safe environment for patients and healthcare workers.

## Figures and Tables

**Table 1:** Cycling conditions and primer sequences for genes presented in this study.

	Description	Gene	Primer sequence	PCR conditions	bp size	References
Species-specific gene	Thermonuclease	*nuc*	F-GCGATTGATGGTGATACGGTT R-AGCCAAGCCTTGACGAACTAAAGC	30 s 95°C, 30 s 55°C, 1 min 72°C	270	[19]

Virulence genes	Haemolysin	*hla*	F-CTGATTACTATCCAAGAAATTCGATTG R-CTTTCCAGCCTACTTTTTTATCAGT	30 s 94°C, 30 s 55°C, 1 min 72°C	209	[20]
		*hld*	F-AAGAATTTTTATCTTAATTAAGGAAGGAGTG R-TTAGTGAATTTGTTCACTGTGTCGA	30 s 94°C, 30 s 55°C, 1 min 72°C	111	[20]
	Panton–Valentine leukocidin	*LukS/F-PV*	F-ATCATTAGGTAAAATGTCTGGACATGATCCA R-GCATCAAGTGTATTGGATAGCAAAAGC	30 s 95°C, 45 s 60°C, 1 min 72°C	443	[20]

Resistance genes	Erythromycin	*ermC*	F-CTTGTTGATCACGATAATTTCC R-ATCTTTTAGCAAACCCGTATTC	30 s 94°C, 30 s 55°C, 1 min 72°C	190	[21]
	Tetracycline	*tetK*	F-TCGATAGGAACAGCAGTA R-CAGCAGATCCTACTCCTT	30 s 94°C, 30 s 55°C, 1 min 72°C	169	[21]
		*tetM*	F-GTGGACAAAGGTACAACGAG R-CGGTAAAGTTCGTCACACAC	50 s 95°C, 1 min 55°C, 1 min 72°C	657	[22]
	Aminoglycosides	*aac* (6′)*-aph* (2″)	F-TAATCCAAGAGCAATAAGGGC R-GCCACACTATCATAACCACTA	30 s 94°C, 30 s 55°C, 1 min 72°C	227	[21]
	Methicillin	*mecA*	F-AACAGGTGAATTATTAGCACTTGTAAG R-ATTGCTGTTAATATTTTTTGAGTTGAA	30 s 94°C, 30 s 55°C, 1 min 72°C	174	[21]
	*β*-Lactamase	*blaZ*	F-ACTTCAACACCTGCTGCTTT R-TGACCACTTTTATCAGCAAC	30 s 94°C, 30 s 55°C, 1 min 72°C	173	[21]

## Data Availability

The generated data used to support the findings of this study are included within the article.

## Supplementary Materials

Supplementary Materials Table S1: description of the samples collected from hospitals, wards, and sampling sites. Table S2: Fischer's exact test and Pearson's chi-square test for virulence and resistance genes from eleven frequently touched sites, wards, and hospitals. Table S3: comparison of Pearson's correlation for virulence and resistance genes detected in *S. aureus* environmental isolates from four public sector hospitals.
