# Supplementary material for: Genotypic and Phenotypic Characterizations of Methicillin-Resistant Staphylococcus aureus (MRSA) on Frequently Touched Sites from Public Hospitals in South Africa
Source: Int J Microbiol. 2021 Oct 23;2021:6011045. doi: 10.1155/2021/6011045 (PMC8556974; doi:10.1155/2021/6011045)
Supplement: Supplementary Materials — Table S1: description of the samples collected from hospitals, wards, and sampling sites. Table S2: Fischer's exact test and Pearson's chi-square test for virulence and resistance genes from eleven frequently touched sites, wards, and hospitals. Table S3: comparison of Pearson's correlation for virulence and resistance genes detected in S. aureus environmental isolates from four public sector hospitals. [file 6011045.f1.docx]

**Supplementary materials**

**Table S1:** Description of the samples collected from hospital, wards and sampling sites

| **Hospitals classification** | **Hospital wards** | **Sites** |
| --- | --- | --- |
| **A**   - Central with tertiary care level-3 facility and known as a referral hospital. - Large as consisted of approximately 800 beds - Provides training for healthcare providers and conduct research. - Central quaternary care hospital. - It provides comprehensive healthcare services that include sophisticated diagnostics and treatment services - Render sub-specialised services include advanced trauma care organ transplantation and have technological complexes equipment and clinical support services. | - General ward - Intensive Care Unit - Paediatrics | - BP monitor - Door handle - Drip stand - Mop - Nurses table - Occupied bed - Unoccupied bed - Patient file - Phone - Sink - Ventilation |
| **B**   - Central with services rendered through the level-3 facility and providing regional and tertiary services to the whole of KZN and Eastern Cape. - Medium-sized hospital with approximately 900 beds. - Also, a teaching hospital. - Provide highly specialised staff and healthcare facilities |  |  |
| **Continue Table S1:** Description of the samples collected from hospital, wards and sampling sites | | |
| **Hospitals classification** | **Hospital wards** | **Sites** |
| **C**   - Regional and formerly classified as district and classified as a level-2 hospital. - It operates 24 hours with trauma and emergency services. - Approximately 500 beds. - Receive support from tertiary hospitals - Limited to province boundaries. - Renders services that can be provided by general specialists in general surgery, diagnostic radiology, pathology and alien health services |  |  |
| **D**   - District hospital, a level-1 facility that has 24-hours trauma services. - Receives referral from several districts’ hospitals. - Consist of 500 beds. - Provides services such as in-patients and emergency health services. - Services are provided by general medical practitioners including surgical interventions under anaesthesia. |  |  |

Table S2: Fischer’s exact test and Pearson’s Chi-Square test for virulence and resistance genes from eleven frequently touched sites, wards and hospitals

|  |  | Asymptotic significance (2-sided) | | | | | | | | |
| --- | --- | --- | --- | --- | --- | --- | --- | --- | --- | --- |
|  | **Statistical Tests** | **Resistance Genes** | | | | | | **Virulence Genes** | | |
|  |  | *mecA* | *tetM* | *tetK* | *ermC* | *aac (6’)-aph (2’’)* | *blaZ* | *LukS/F-PV* | *hla* | *hld* |
| Site | Pearson’s Chi-Square Tests | **0.031*** | 0.831 | 0.06 | 0.255 | 0.887 | 0.590 | 0.577 | 0.185 | 0.361 |
|  | Fisher’s exact test | **0.02*** | 0.825 | **0.05*** | 0.215 | 0.916 | 0.639 | 0.646 | 0.170 | 0.424 |
| Ward | Pearson’s Chi-Square Tests | 0.158 | 0.831 | 0.062 | 0.255 | 0.887 | 0.590 | 0.577 | 0.185 | 0.361 |
|  | Fisher’s exact test | 0.125 | 0.054 | 0.163 | 0.761 | 0.375 | 0.067 | 0.107 | 0.314 | 0.362 |
| Hospital | Pearson’s Chi-Square Tests | 0.124 | 0.057 | **0.000*** | **0.010*** | **0.003*** | 0.111 | **0.000*** | 0.191 | 0.061 |
|  | Fisher’s exact test | 0.104 | 0.051 | **0.000*** | **0.008*** | **0.003*** | 0.116 | **0.000*** | 0.196 | 0.072 |

The statistical significance of the relationship between the genes detected and the site, ward and hospital were examined. The highlighted values with an asterisk (*) indicated a probability value of less than 0.05 *(p < 0.05).*

Table S3: Comparison of Pearson’s correlation for virulence and resistance genes detected in *S. aureus* environmental isolates from four public sector hospitals

|  | | | | | | | | | | |
| --- | --- | --- | --- | --- | --- | --- | --- | --- | --- | --- |
|  | | mecA | tetK | ermC | blaZ | aac (6’) aph (2") | hla | hld | tetM | lukS/F-PV |
| mecA | Pearson Correlation | 1 | .255^*^ | .360^**^ | .225^*^ | .216^*^ | -.098 | -.013 | .416^**^ | .225^*^ |
|  | Sig. (2-tailed) | - | .011 | .000 | .025 | .032 | .335 | .898 | .000 | .025 |
| tetK | Pearson Correlation | .255^*^ | 1 | .307^**^ | .102 | .283^**^ | -.243^*^ | .084 | .354^**^ | .348^**^ |
|  | Sig. (2-tailed) | .011 | - | .002 | .316 | .005 | .016 | .411 | .000 | .000 |
| ermC | Pearson Correlation | .360^**^ | .307^**^ | 1 | .472^**^ | .333^**^ | -.126 | .054 | .368^**^ | .391^**^ |
|  | Sig. (2-tailed) | .000 | .002 | - | .000 | .001 | .215 | .596 | .000 | .000 |
| blaZ | Pearson Correlation | .225^*^ | .102 | .472^**^ | 1 | .377^**^ | -.085 | .054 | .202^*^ | .188 |
|  | Sig. (2-tailed) | .025 | .316 | .000 | - | .000 | .403 | .596 | .045 | .063 |
| aac (6’) aph (2") | Pearson Correlation | .216^*^ | .283^**^ | .333^**^ | .377^**^ | 1 | -.186 | -.194 | .246^*^ | .199^*^ |
|  | Sig. (2-tailed) | .032 | .005 | .001 | .000 | - | .065 | .055 | .014 | .048 |
| hla | Pearson Correlation | -.098 | -.243^*^ | -.126 | -.085 | -.186 | 1 | .068 | -.219^*^ | -.166 |
|  | Sig. (2-tailed) | .335 | .016 | .215 | .403 | .065 | - | .505 | .029 | .100 |
| hld | Pearson Correlation | -.013 | .084 | .054 | .054 | -.194 | .068 | 1 | .041 | .184 |
|  | Sig. (2-tailed) | .898 | .411 | .596 | .596 | .055 | .505 | - | .688 | .070 |
| tetM | Pearson Correlation | .416^**^ | .354^**^ | .368^**^ | .202^*^ | .246^*^ | -.219^*^ | .041 | 1 | .285^**^ |
|  | Sig. (2-tailed) | .000 | .000 | .000 | .045 | .014 | .029 | .688 | - | .004 |
| lukS/F-PV | Pearson Correlation | .225^*^ | .348^**^ | .391^**^ | .188 | .199^*^ | -.166 | .184 | .285^**^ | 1 |
|  | Sig. (2-tailed) | .025 | .000 | .000 | .063 | .048 | .100 | .070 | .004 | - |
| *. Correlation is significant at the 0.05 level (2-tailed) and indicates significance of gene association | | | | | | | | | | |
| **. Correlation is significant at the 0.01 level (2-tailed) and indicates significance of gene association | | | | | | | | | | |
